# Integrated primary health care in low- and middle-income countries: a double challenge

**DOI:** 10.1186/s12910-018-0288-z

**Published:** 2018-06-15

**Authors:** Thomas Druetz

**Affiliations:** Department of Tropical Medicine, Tulane University, 1440 Canal St, New Orleans, LA USA

**Keywords:** Primary health care, Integrated health services, Aid coordination, Health system integration, Public health system strengthening

## Abstract

**Background:**

The lack of primary healthcare integration has been identified as one of the main limits to programs’ efficacy in low- and middle-income countries. This is especially relevant to the Millennium Development Goals, whose health objectives were not attained in many countries at their term in 2015. While global health scholars and decision-makers are unanimous in calling for integration, the objective here is to go further and contribute to its promotion by presenting two of the most important challenges to be met for its achievement: 1) developing a “crosswise approach” to implementation that is operational and effective; and 2) creating synergy between national programs and interventions driven by non-State actors.

**Main body:**

The argument for urgently addressing this double challenge is illustrated by drawing on observations made and lessons learned during a four-year research project (2011–2014) evaluating the effects of interventions against malaria in Burkina Faso. The way interventions were framed was mostly vertical, leaving little room for local adaptation. In addition, many non-governmental organizations intervened and contributed to a fragmented and heteronomous health governance system. Important ethical issues stem from how interventions against malaria were shaped and implemented in Burkina Faso. To further explore this issue, a scoping literature review was conducted in August 2016 on the theme of integrated primary healthcare. It revealed that no clear definition of the concept has been advanced or endorsed thus far. We call for caution in conceptualizing it as a simple juxtaposition of different tasks or missions at the primary care level. It is time to go beyond the debate around selective versus comprehensive approaches or fragmentation versus cohesion. Integration should be thought of as a process to reconcile these tensions.

**Conclusions:**

In the context that characterizes many low- and middle-income countries today, better aid coordination and public health systems strengthening, as promoted by multisectoral approaches, might be among the best options to sustainably and ethically integrate primary healthcare interventions.

## Background

Despite many achievements, there was growing concern that the Millennium Development Goals (MDGs) would not be attained by 2015 in several low- and middle-income countries (LMICs). A major issue stemmed from the growing complexity of global health governance [1]. In particular, the large increase in funding, notably thanks to private philanthropists and international initiatives, supported or introduced numerous disease-specific programs and selective interventions [2]. In many LMICs, the already fragile health systems were not adequately strengthened, which limited their capacity to implement such health initiatives [3, 4].

In preparation for the post-MDGs era, the World Health Organization (WHO) examined these difficulties and the main obstacles that impeded countries’ efforts to achieve the MDGs. In 2008, WHO published a technical report on the lack of health services integration, consistently identified as one of the main limitations to programs’ efficacy in LMICs. Based on that examination, WHO suggested a major change in global health strategies to face this challenge, with the 2008 World Health Report entitled *Primary Health Care: Now More Than Ever*. The new orientation towards “integrated primary health care” was formally adopted the following year at the 62nd World Health Assembly and was recently detailed and re-emphasized in the 2015 WHO *Global Strategy on People-Centered and Integrated Health Services*. This represented a major undertaking and not a mere adjustment—it was a call for a “fundamental paradigm shift in the way health services are funded, managed, and delivered” [5].

In the following pages, the argument is made that integrating primary health programs into the health systems of LMICs faces two major hurdles. The first is a growing tendency to fund interventions that are vertical, selective, and disease-oriented, as opposed to horizontal, comprehensive, and system-wide. The integrated model is viewed as a dialectic that would reconcile this long-standing opposition between horizontal and vertical approaches, but it remains to be operationalized. The second is the fragmented health governance that characterizes many LMICs. Indeed, non-governmental organizations (NGOs) have a predominant role in the health sector and often implement interventions on their own, especially at the local level, which sometimes may thwart national health programs or strategies.

Consequently, the double challenge that characterizes the integration agenda can be defined as the urgent need: 1) to find and develop a “cross-cutting approach” that is operational and effective, and 2) to coordinate efforts in the health sector to overcome the tension between national programs and local NGO-driven interventions. While global health scholars and decision-makers are calling unanimously for integration, the objective in this article is to go further and contribute to its promotion by presenting these two important challenges that need to be addressed. To illustrate this double challenge, I draw on lessons from my PhD research in Burkina Faso, where I evaluated the implementation and effectiveness of a nation-wide program against malaria, called “community case management of malaria”. I will present how this particular program encountered issues related to this double challenge, how it was affected by them, and how it raised important ethical issues. This article is also innovative in advancing a refined conceptual framework in which integration is conceptualized as a process to balance two tensions: 1) vertical vs. horizontal approaches; and 2) cohesive (state-centred) vs. fragmented health governance.

## Main text

### Primary health care as a call for equity

In the 1960s and 1970s, many LMICs started criticizing the occidental medico-centred health systems inherited from the colonial era. These disease-oriented systems mostly promoted inequities, since they were focused on providing care to the urban elite and, to some extent, to the workers of large companies [6, 7]. They ignored the basic health needs of the population. In Western countries, experts and scholars also began to question the biomedical paradigm and the belief that better population health would result from economic development in a trickle-down effect [8–11]. Population health became an issue of justice and social development. It demanded policies and programs specifically dedicated to improving the living conditions of the entire population.

At the same time, LMICs in the Third World (non-aligned) or associated with the Soviet bloc experimented with new approaches to improve the health of their populations. An example is the Chinese Barefoot Doctors Program, which received considerable attention. This national program consisted of training village workers, mostly farmers, who would provide preventive and curative health care services to members of their community [12]. Other community programs to improve the health of rural populations were implemented in Venezuela, Tanzania, India, and Yugoslavia. [13, 14]. The Cuban experience was also studied. Before the 1959 revolution, the health system was mainly private, with physicians concentrated in large cities and most rural populations having little access to health services. Within a decade, the revolutionary regime had completely reorganized the health system (including a gradual nationalization), made health care free, decentralized outpatient primary health care (PHC), fully engaged communities in health planning, and adopted targeted vertical programs and broad interventions on the social determinants of health (education and sanitation) simultaneously [15]. In 1975, nearly 100% of the population had access to health care, coverage of many programs was above 90%, and health indicators revealed a remarkable improvement of the health of Cubans [16].

State delegates to the World Health Assembly discussed these new and alternative approaches. Starting in the late 1960s, WHO promoted several projects to improve first-line health services in LMICs. In 1972, these efforts gave rise to a new WHO department—Strengthening of Health Services. Finally, in a remarkable (but short-lived) partnership, WHO and United Nations Children’s Fund (UNICEF) held the Alma-Ata Conference, at which the PHC policy was consensually adopted by the international community. All 134 participating countries signed the Declaration at the end of the conference in September 1978.

### From comprehensive to selective primary health care

The pillars of the PHC policy have been abundantly examined in the scientific literature: holistic definition of health, decentralization, multisectoral development, community participation, individual empowerment, social change, prevention, and justice [17–21]. While basic health care (i.e., care for minor or easy-to-treat health problems) was also included in PHC, this policy’s primary objective was to contribute to communities’ social development and self-determination and consequently it was conceptualized as a comprehensive enterprise. Signatories committed themselves to “formulate national policies, strategies and plans of action to launch and sustain primary health care as part of a comprehensive national health system and in coordination with other sectors” [22]. Strategies adopted after Alma-Ata sometimes affected numerous sectors simultaneously: health, education, agriculture, engineering, environment, landscaping and country planning, hygiene, and local economic development [23]. Years before academics advanced today’s cornerstone concepts in public health (i.e., health promotion, social determinants of health), the PHC policy already suggested adopting an ecological and comprehensive perspective on health [24].

Despite the apparent agreement, the PHC policy has suffered from criticism since its inception. The Alma-Ata Declaration has been described as an empty policy document with an unrealistic goal and good intentions but no clear program or budget allocation [13]. It has also been argued that its emancipatory message was not in line with Western countries’ desire to control the path to health development, and the PHC policy was pegged as a communist policy [25]. Donors concerned about corruption were reluctant to lose control of the funds and demanded that their experts or advisors supervise the programs. In the same vein, the principles of decentralization and community participation in decision-making faced considerable resistance, while changes in the global political and socio-economic context undermined the ability to introduce the systemic transformation suggested at Alma-Ata [13, 26, 27].

Shortly after Alma-Ata, international organizations and scholars invited by the Rockefeller Foundation suggested re-orienting the PHC policy towards a more vertical, disease-centred approach [6]. Now referred to as *selective* PHC, this strategy promoted a small number of technical interventions against the most prevalent diseases and health problems in LMICs. It rapidly restored the focus on vertical programs (allegedly more cost-effective than a comprehensive program) and the role of medical experts among them [28, 29]. In defining a few key diseases that programs should specifically target, the selective PHC policy contradicted the core principle of PHC; in that sense, “selective PHC” effectively became an oxymoron. This perfectly illustrates the tension between vertical and horizontal approaches in PHC [30].

### Cohesive vs. fragmented primary health care

The Alma-Ata Declaration stated that PHC “…forms an integral part both of the country’s health system, of which it is the central function and main focus, and of the overall social and economic development of the community” [22]. It attributed a central role to the State, as the actor in charge of developing and implementing intersectoral policies to improve population health. Even within the health sector, this intention led to the defining of two key missions: 1) to integrate the missionary and private dispensaries that provide front-line health services in rural areas into the public body, and 2) to strengthen this new public health system.

In that sense, PHC is intrinsically cohesive; it calls for efforts that are public and under the primary responsibility of the State. State interventionism is a key feature of the spirit prevailing at the Conference. Again, this concern stemmed from an ethical position: LMICs had to intervene to reduce health and social inequities. Some countries—the USSR in particular—pushed for centralized PHC, while others, including Brazil and several countries in Southeast Asia and Latin America, conceived of it as empowerment and decentralized policy [31]. While this question of power devolution to local authorities and communities remained unclear at the time, it was widely accepted that the Declaration called for State-driven enterprise.

However, the neoliberal ideology of the 1980s directly opposed this approach. Advancing an agenda focused on reduction of government expenditures, free markets, privatization, and marketization, multilateral institutions such as the International Monetary Fund and the World Bank exerted pressures to restrain State-driven actions in many sectors, including health. Structural adjustment programs and the Bamako Initiative are some of the best-known illustrations of these pressures. This paved the way to a dramatic proliferation of NGOs [1, 32].

The health sector was particularly affected [33]. For the past 30 years, foreign health aid has been massively channelled through NGOs implementing local or national interventions [3]. This was accelerated by the establishment of philanthropist foundations and public/private initiatives that are now key donors in the health sector [34]. This “aid industry” [35] is a fundamental element of the fragmented health governance that today characterizes many LMICs. It is noteworthy that this turbulence phenomenon [36] does not occur solely at managerial or central levels; PHC fragmentation has been observed in a variety of contexts [37, 38].

### Vertical and fragmented: a matter of ethics

After decades of pressure towards privatization and neo-liberalization in LMICs, experts started to realize that the new “complexity” of health systems entailed new ethical issues [29]. Increased funding by private donors disempowered LMICs, whose control of both agenda-setting and the implementation of public health interventions decreased correspondingly. It also raised the issue of accountability: who is responsible for interventions funded by foreign donors and implemented by international NGOs? These problems not only contribute to the demise of Nation States, but can also undermine efforts to improve population health [39]. For example, contradictory health-related messages from a multiplicity of actors, each with their own priorities and agenda, can confuse the population. Also, if local health systems are not strengthened, external interventions have only limited effectiveness, especially in remote areas where the most vulnerable populations live [40]. In countries with limited resources and huge needs, the efficiency of interventions encompasses a critical ethical dimension.

In this context, the Paris Declaration on Aid Effectiveness was adopted in 2005 to address the ethical challenges and promote five principles: ownership, alignment, harmonization, results-based management, and mutual accountability. As outlined by Lavigne Delville [39], these principles are aimed mainly at mitigating the adverse effects of the multiplicity of donors and the heteronomy of States.

### Interventions against malaria in Burkina Faso

As explained above, while PHC was designed to be a horizontal, comprehensive, and cohesive policy to reduce health inequities, it has rapidly been reframed as a vertical and fragmented approach to implement selective interventions against specific diseases. The situation in Burkina Faso, where I have been doing fieldwork for the past six years, is illustrative of this challenge. Burkina Faso is one of the countries with the highest malaria (*P. falciparum*) prevalence [41]. In this country of 16 million inhabitants, approximately 40,000 deaths are attributed every year to malaria [42]. It is the first cause of morbidity and mortality in children under five, and the primary reason for nearly half of the consultations at health centres [43]. Malaria is arguably one of the most important public health issues in Burkina Faso.

Data were collected during five trips to Burkina Faso between May 2011 and March 2014. Over this period, I was involved in a research project to evaluate the implementation and effectiveness of a program called “community case management of malaria” that Burkina Faso had scaled up nationally in 2010 [44]. For this research, which comprised quantitative and qualitative components, I conducted two extensive periods (three and four months) of fieldwork in the district of Kaya. The study was conducted in this district thanks to the collaboration of a local Demographic and Health Observatory.

Field immersion was very rich and included non-participant observation of treatment-seeking practices, informal discussions about the malaria case management strategy with program planners in the capital (Ouagadougou) and with local stakeholders, and semi-structured interviews with community health workers (*n* = 17), nurses in health facilities (*n* = 6), NGO representatives (*n* = 3), district health authorities (*n* = 4), and caregivers in the communities (*n* = 31). Interviews with nurses and health authorities were repeated in 2011 and 2013. Field notes were systematically taken and recorded in research logbooks and analyzed using an inductive approach to explore themes related to health governance and integrated interventions. The study was approved by the research ethics committees in Burkina Faso and at the University of Montreal Hospital Research Centre. Participation in the study was not remunerated, and informed consent was obtained from every participant, as required by the ethics committees.

The way efforts against malaria are framed in Burkina Faso is similar to what is done in other countries. The National Program against Malaria is theoretically in charge of coordinating all efforts against malaria. A large portion of its work, however, involves submitting proposals to the funding organizations, usually the Global Fund to Fight AIDS, Tuberculosis and Malaria. To qualify for funding, submissions advance interventions that fit the recommendations set up by international health organizations (WHO or Roll Back Malaria). To facilitate this, foreign consultants are sometimes hired or experts from well-known agencies are delegated to format the submissions. The entire process is vertical: (1) key interventions are recommended by a partnership specifically dedicated to malaria (Roll Back Malaria); (2) these recommendations are endorsed by international funding agencies; (3) countries prepare submissions that integrate these malaria-specific interventions; and (4) the National Program against Malaria is in charge of implementing, monitoring, and reporting on activities. The entire system is pushing for vertical interventions against malaria [29, 45].

The nationwide, community-based program against malaria introduced in 2010 in Burkina Faso received financial support from the Global Fund. Most of the budget was allocated to two malaria-specific activities: using community health workers (CHWs) to administer treatments and distributing bed nets. No other sector was included in developing or implementing the program. While it has been repeatedly acknowledged that malaria is the expression of a social, economic, environmental, and biological vulnerability [46], the distal and multisectoral determinants of the disease were ignored in this strategy. Instead, the program was embedded in a biomedical paradigm that today governs primary health care in LMICs [47]. Millions of bed nets were distributed in communities, but the living environment of the population remained mostly unchanged. *Anopheles* are still omnipresent today and tend to bite earlier in the evening, when people are less likely to be protected by bed nets. CHWs received training to administer age-appropriate treatment dosages, but were often illiterate and could not read the refresher pamphlets. Regimens for adults were given to small children, because the drug packages had similar colours. Children under five received free seasonal chemoprevention treatments, but a large proportion of their parents still believed malaria to be “the disease of the bird” and that tree barks were an effective form of medication that can sometimes be combined with, or replace, modern treatment [48].

Perhaps even more striking was how fragmented the efforts against malaria were in the country. Concurrently with the above-mentioned strategy, several NGOs were employing CHWs to do the same thing, i.e., administer malaria treatment in the villages. Some NGOs distributed bed nets for pregnant women or young children; others disseminated sensitization messages, and yet others conducted rapid diagnostic testing, and so on. According to Kaya district health authorities, there were more than 80 NGOs working in the field of malaria in the district. It was among the NGOs’ favourites because it was close to the capital and roads were good. It was impossible for health authorities to coordinate the efforts against malaria.

This fragmentation generated numerous problems. Non-participant observation and interviews with CHWs revealed that many of them could no longer remember which (or how many) organizations had recruited them, or the target population(s) to whom they were supposed to administer treatments [49]. Some NGOs were disseminating messages encouraging febrile individuals to consult the nearest health centre; meanwhile others were introducing community case management and advising the rural population to visit a CHW in their village in case of fever. One NGO supported the removal of user fees for visits to health centres, but not to CHWs; patients still had to pay for treatments when consulting CHWs [50]. This completely undermined community case management strategies—including the above-mentioned national program introduced by health authorities—in the entire district [51, 52].

Important ethical issues stem from this situation. Contradictory recommendations and messages confused treatment-seeking practices among the population, and precious resources were wasted in a very necessitous environment—Burkina Faso ranks 183rd of 188 countries in the Human Development Index [53]. Communities’ trust towards health actors (NGOs, CHWs, nurses, etc.) was damaged, and an already present feeling of disillusionment was galvanized. Nurses in PHC centres were not consulted before the introduction of uncoordinated interventions in their catchment area and resented this new competitive, commodified health environment. At the district level, NGOs continued to bypass the health authorities, even though both were aware of the problems created by fragmented and heteronomous health governance. These tensions are symptomatic of unequal relations between the main actors fighting malaria in Burkina Faso, an inequity that is inherent to the verticalization and fragmentation of PHC.

## Conclusions

### What would (and would not) be integrated primary health care?

My intention here is not to categorically reject the PHC model now prevalent in LMICs or to imply that what has been done in the past 30 years is fundamentally wrong. The other ideal-type (the original PHC model) was not free of pitfalls and issues [54]. Rather than calling for a new “Alma-Ata revolution”, I argue that a dialectic is needed to reconcile the tensions between (i) horizontal and vertical approaches and (ii) cohesive and fragmented models of governance. This dialectic should govern the definition of integrated PHC—a concept for which no definition of has been advanced until now.

Unfortunately, integrated PHC is often mistakenly understood as a juxtaposition of different tasks or missions at the primary care level. When CHWs administer treatment for a package of three or four diseases (usually malaria, pneumonia, diarrhea, and malnutrition) instead of only one, many authors refer to this as an integrated strategy—or even as integrated PHC [55–57]. This confusion has been facilitated by the WHO and UNICEF endorsement in 2012 of the integrated community case management strategy, which is nothing more than a combined management of several diseases by CHWs [58]. Combining several vertical interventions is arguably a step in the right direction [59], but integration is more than that.

I advance here that two key mechanisms must be triggered in LMICs for PHC to become integrated. The first is coordination of aid and health interventions, which should apply not only to the interventions’ content, but also to their implementation [60]. Coordination is essential at all levels of implementation and should be under the responsibility of the recipient State; in many LMICs, decentralized public coordination organs are urgently needed. Partnerships between NGOs and health authorities are an interesting option to better involve State actors and increase accountability. However, partnerships can be affected by power inequalities and do not preclude the implementation of vertical or isolated interventions. Several coordination mechanisms already exist and, in some countries, have proven to be effective, such as sector-wide approaches that bring together governments, donors, and stakeholders within any sector to develop interventions [61, 62]. While coordination has been recognized for a decade as a focal challenge in the field of development—it was one of the core principles of the Paris Declaration [63]—there is considerable reluctance to fully adopt effective mechanisms, especially at the local level [64, 65].

The second essential mechanism for achieving integrated PHC is *public health* systems strengthening. This builds upon the concept of health systems strengthening, which has been widely debated in recent years and calls for interventions that systematically help to make health institutions and systems sustainable and well-functioning [66]. In the same vein, integrated PHC requires long-term capacity-building in the health sector—again, at both the central and local levels [2]. Integration also involves, however, applying a positive and inclusive definition of health and considering the distal determinants of health when designing interventions [5]. As such, in this article I call for *public health* system strengthening, to draw attention to the fact that a multisectoral and multidisciplinary perspective is intrinsically linked to integrated PHC [67]. This would entail, for example, stepping outside the logic of sector-wide approaches and inviting stakeholders from different sectors into the decision-making process. Such multisectoral approaches are currently being tested as a framework for several health interventions, including the most recent UNICEF projects to reduce malnutrition or to improve water, sanitation, and hygiene (WASH) [68–70]. This is a promising mechanism to link multisectoral actions, rather than providing only medical and disease-oriented services.

The intention here has not been to present an exhaustive review of the challenges standing in the way of integrated PHC, nor to delineate solutions to meet these challenges or to resolve decades-long debates about health systems organization in LMICs. Rather, the aim of this article is simply to highlight problems that can be caused by the “verticalization” of programs and the fragmentation of health governance. As proposed by Marchal et al., it is time to go beyond the debate between selective versus comprehensive approaches, or fragmentation versus cohesion [66]. The main argument here is that integration can be thought of as a process to reconcile these tensions. In the context that characterizes many LMICs today, better aid coordination and the strengthening of public health systems—as multisectoral approaches try to promote—might be among the best options to integrate PHC interventions sustainably and ethically.

## Abbreviations

CHW: Community health worker
LMICs: Low- and middle-income countries
MDG: Millennium Development Goals
NGOs: Non-governmental organizations
PHC: Primary healthcare
UNICEF: United Nations Children’s Fund
USSR: Union of Soviet Socialist Republics
WHO: World Health Organization
